# Tuning the value of sweet food: Blocking sweet taste receptors increases the devaluation effect in a go/no-go task

**DOI:** 10.3758/s13423-025-02666-w

**Published:** 2025-02-25

**Authors:** Toni Cunillera, Neus Nuño, Marc Ballestero-Arnau, Borja Rodríguez-Herreros, Cristina Rodríguez-Jiménez, Mercè Pallàs

**Affiliations:** 1 Department of Cognition, Development and Educational Psychology, Faculty of Psychology, Universitat de Barcelona, Pg. Vall d’Hebron 171, 08035 Barcelona, Spain; 2 Institute of Neurosciences (UBNeuro), Universitat de Barcelona, 08035 Barcelona, Spain; 3 Service Des Troubles du Spectre de L’Autisme Et Apparentés, Centre Hospitalier Universitaire Vaudois, 1011 Lausanne, Switzerland; 4 Department of Pharmacology, Toxicology and Therapeutic Chemistry, Faculty of Pharmacy, Universitat de Barcelona, Campus Diagonal, Joan XXIII, 27-31, 08028 Barcelona, Spain

**Keywords:** Go/no-go, Gymnemic acid, Value-updating, Inhibitory function, Macronutrients

## Abstract

Despite the apparent simplicity of the go/no-go (GNG) task, in which individuals selectively respond or withhold responses, there is strong evidence supporting its efficacy in terms of modulating food preferences. Herein, we manipulated sweet taste perception and investigated the no-go devaluation effect that is typically observed due to GNG training with respect to sweet and savory food items. Prior to engaging in a GNG task, one group of participants rinsed their mouths with a liquid solution containing gymnemic acid, thereby transiently and selectively inhibiting sweet taste perception, while another group used a placebo solution. The participants who rinsed their mouths with gymnemic acid exhibited a stronger overall decrease in food evaluations from pre to post training. Furthermore, a pronounced no-go devaluation effect was observed for sweet foods, irrespective of the rinsing solution. Overall, our results support the notion that training in the GNG task can induce changes in the valuation of food stimuli, particularly for sweet foods.

**Supplementary Information:**

The online version contains supplementary material available at 10.3758/s13423-025-02666-w.

## Introduction

In our contemporary obesogenic environment, which is characterized by the ubiquity of ultra-processed foods, public health interventions to improve dietary intake have failed to curb the consumption of these food types (Hawkes et al., 2015). Accordingly, there has been a steady increase in obesity rates in recent decades (Di Cesare et al., 2016). Although people usually have difficulties with resisting the temptation to consume unhealthy foods and changing their eating habits, it has been shown that food preferences are malleable throughout the lifespan (Ventura & Worobey, 2013).

The ability to resist temptation is inherently aligned with the concept of self-control, which in turn has been associated with inhibitory brain function (Diamond, 2013). It has been consistently proven that simple repetitive action or inaction toward different types of food stimuli – in the absence of any external reinforcement – is effective in inducing both food consumption and food preference changes (McGreen et al., 2024a). Two promising methods to modulate food preferences have sparked interest in recent years: the food go/no-go (GNG) task and the cue-approach training task (Houben & Aulbach, 2023). While it is widely accepted that changes in food preferences induced by cue-approach training are due to alterations in perceptual and attentional mechanisms (Botvinik-Nezer et al., 2020; Schonberg & Katz, 2020), the cognitive mechanisms underlying the food devaluation effect induced by GNG training remain unclear (Chen et al., 2016; Veling et al., 2022). GNG training has been proposed to promote inhibitory control and consequently reduce the impulse to eat unhealthy foods through repetitive pairings of food cues with inaction responses, thereby resulting in the devaluation of the associated food items (Houben & Aulbach, 2023; Veling et al., 2017, 2022). This leads to a subsequent reduction in the intake and choice of those foods (Aulbach et al., 2019; McGreen et al., 2024a; Yang et al., 2019). Furthermore, significant weight loss has been observed in participants who undertook GNG training with unhealthy food items used as no-go-trained items (Houben & Jansen, 2015; Yang et al., 2019).

Until recently, the prevailing explanation of such a devaluation effect has suggested a causal contribution of inhibitory brain function (Tzavella & Chambers, 2023). Essentially, the association between visual stimuli and response inhibition during a no-go trial is formed and reinstated again in subsequent no-go trials (Chen et al., 2016; Goolsby et al., 2009), thus leading to simultaneous item devaluation through several putative pathways involving inhibitory function (see Veling et al., 2022). Despite the different explanations attributing a major role to the inhibitory function, the interpretation of these results seems to be aligned with the view that the inhibitory function has a prominent role in modulating food-related self-control behaviors (Houben & Aulbach, 2023; Veling et al., 2022). Nevertheless, an alternative theoretical perspective has emerged within the framework of value-based decision-making (Rangel et al., 2008), with no need to claim the involvement of an inhibitory function to explain the devaluation process. This value-updating account posits that devaluation effects – as well as related changes in food preferences – arise from alterations in the value of food items following training with the GNG task. Similarly, Berkman and coauthors (2017) proposed a model to explain self-control as a value-based choice in which decision-making is achieved through the dynamic integration of the subjective value assigned to each possible decision. Interestingly, there is no need to incorporate any inhibitory function in this model.

More specifically, value-updating theory posits that repeated inaction decisions in no-go trials automatically lead to a decrease in the value of these associated cues (Veling et al., 2022). This no-go devaluation effect is understood as the result of an automatic readjustment in the given value of rewarding stimuli following inaction responses prompted by a no-go signal presented shortly after the rewarding stimulus. It is assumed that a rewarding food stimulus inherently elicits approach behavior – consistent with a go response – due to previously established Pavlovian associations. Consequently, the conflict generated by the sequential presentation of approach-avoidance orders, which occurs when a no-go signal is introduced shortly after a satisfying stimulus cue, can be resolved by attenuating the response bias. This attenuation is then reflected in the devaluation of the no-go stimulus involved.

In the present study, we aimed to assess the validity of the value-updating hypothesis to explain the nature of the devaluation effect that is typically induced by GNG training. To achieve this goal, we conducted a double-blind study in which two groups of participants performed a cue-GNG task: the first group rinsed their mouths with a solution of gymnemic acid (GA), which is a substance that is known to selectively block sweet receptors temporarily (Sanematsu et al., 2014); the second group of participants rinsed their mouths with a placebo solution consisting of black tea mixed with gentian root. Both solutions were carefully designed to be equivalent in taste, color, and astringency. Additionally, we used a large set of appetized food images categorized into two conditions based on the primary macronutrient present: sweet foods (with carbohydrates in the form of glucose and fructose) and savory foods (high in protein and fats). By blocking the gustatory pathway of sweet taste perception with GA, we aimed to disrupt the neural circuit involved in sweet reward processing (Stice & Yokum, 2018). We hypothesized that blocking this pathway would alter the perception of the hedonic value attributed to sweet foods and thus reduce the expected level of pleasantness experienced when assessing the appeal of food images (Stice & Yokum, 2018; Stice et al., 2017). Following the paradigm developed by Chen et al. (2016), participants were exposed to a series of food images and asked to rate their appeal on two separate occasions. The first assessment took place at the beginning of the experiment, and the second assessment was conducted after completing the rinsing procedure and the GNG task.

Our first aim was to replicate the devaluation effect observed in the GNG task, where the appeal of stimuli in the no-go condition is rated lower than that in the go and untrained conditions (Chen et al., 2016). Our second aim was to determine whether the largest devaluation effect observed for the no-go items could be explained in terms of either value updating or response inhibition. This aim was addressed by comparing GNG-induced devaluation between the GA and placebo conditions, as well as between the sweet and savory food conditions. Based on the premises of the value-updating account, we expect that most rewarding food images – those initially evaluated as more appealing, whether sweet or savory – would exhibit the greatest devaluation effect when paired with no-go signals during GNG training. Furthermore, considering that GA reduces the reward expectancy of sweet food images, we predict that the decrease in food ratings would be less pronounced for sweet foods than for savory foods. This difference is likely to be due to a substantial reduction in conflict between the elicited approach behaviors and the expected reward value in no-go trials for sweet foods. Statistically, we would expect to find a three-way interaction between training conditions, rinsing solution, and food category. A significant triple interaction would indicate that savory foods in no-go trained items with the placebo rinsing condition would exhibit the strongest degree of devaluation, whereas the lowest degree of devaluation would be observed in sweet foods with the GA rinsing. Conversely, if the devaluation effect is comparable across both food categories and rinsing solutions in the no-go training condition, it would imply that the devaluation is primarily mediated by the inhibitory function, which is expected to act uniformly on all no-go items.

## Method

### Participants

A total of 104 healthy volunteers participated in the study. All participants provided informed consent upon enrollment and received monetary compensation after the end of the experiment. Four participants were excluded because of noncompliance with the stipulated fasting requirements. Three participants were excluded because their performance in the GNG task deviated from the mean by more than 3 *SD*s. The final sample consisted of 45 participants in the GA condition (41 women; mean age: 20.7 years (*SD* = 2.7); mean body mass index (BMI): 21.6 (*SD* = 2.9)) and 52 participants in the placebo condition (40 women; mean age: 21.4 years (*SD* = 2.7); mean BMI: 21.7 (*SD* = 2.5))]. No statistically significant difference was found between the age of GA and placebo participants [*t*(98) = -1.21; *p* = 0.230] or BMI [*t*(98) = -0.14; *p* = 0.890]. Ethical approval for all procedures was granted by the local ethics committee (IRB00003099).

At the end of the experiment, a power analysis was conducted using G*Power (Erdfelder et al., 2009), which indicated that a sample size of 84 participants was sufficient to achieve a power of 95%, with an a priori alpha level set at *p* = 0.05, to detect the effects of two within-subject factors and one between-subject factor, assuming a moderate effect size estimate (*η*_p_^2^ = 0.06).

### Materials

#### Food images

Ninety food pictures were selected from a food image database (Blechert et al., 2014). Of these, 45 images depicted palatable sweet foods, whereas the other 45 images depicted savory foods (see Appendix A in the Online Supplementary Material). Both sets of images were distinguishable by their carbohydrate content per 100 g [sweet foods: 36.55 g; savory foods: 20.59 g; *t*(88) = 3.58; *p* < 0.001], whereas they were equivalent in terms of fat (*p* = 0.911), calories (*p* = 0.313), and total grams (*p* = 0.409). To ensure comparability, both groups of images were carefully balanced across a range of image properties, including spatial frequency, brightness, contrast, object size, recognizability, familiarity, complexity, and valence. Among these properties, palatability [*t*(88) = 3.02; *p* = 0.003], craving [*t*(88) = 2.52; *p* = 0.013], and arousal [*t*(88) = 1.96; *p* = 0.052] could not be balanced and were greater for sweet foods.

#### Liquid solutions

We prepared two different liquid solutions that were carefully matched for taste, astringency, and color through an iterative process of testing various recipes and proportions and piloting short taste-perception tests. A fresh solution containing GA was prepared according to Kurihara (1969). The GA solution contained 5 g/l of *Gymnema sylvestre* extract (25% purity from GA Creative Enzymes, Shirley, NY, USA, Cat. no. EXTW-098) prepared in 0.01 M NaHCO_3_, resulting in a GA concentration of 1.25 g/l. The active blockage of sweet gustatory receptors by GA is specified to last for at least 30 min (Brala & Hagen, 1983; Sanematsu et al., 2014). The placebo solution was freshly prepared with 6 g/l black tea (Twinings of London Prince of Wales) and 7 ml/l gentian decoction (2%) in 0.01 M NaHCO_3_. The tea was used to match both solutions in terms of color and bitterness (Warren et al., 1969), and the gentian root was used to add extra bitterness and astringency to this solution, making it comparable to the GA solution. Both solutions were carefully prepared and labeled by two of the co-authors who were not involved in the data collection process. The labels for each solution remained coded until data collection was completed. Both solutions were stored at 4 °C until use.

### Questionnaires

#### Food consumption questionnaire

We designed an online questionnaire to assess the frequency of food consumption at the time of participant enrollment in the study. The response options included (1) daily, (2) several times a week, (3) once a week, (4) once every 15 days, (5) once a month, (6) rarely, and (7) never. These options were presented alongside the corresponding Spanish food terms or descriptions (e.g., “spaghetti with Bolognese sauce”). The questionnaire items were matched to the food images used in a subsequent stage of the study.

*Sweet taste questionnaire* (STQ; Kampov-Polevoy et al., 2006).

The STQ included a total of 12 items and a 7-point Likert scale ranging from 1 (completely disagree) to 7 (completely agree) to indicate the level of agreement with each item. The STQ was found to assess two distinct factors related to an individual's disposition toward sweet foods: (1) sensitivity to the mood-altering effects of sweet food consumption and (2) impaired control over sweet food consumption. Elevated scores are indicative of increased emotional dependence on sweet foods and a greater degree of difficulty in controlling sweet eating habits, respectively.

#### Liquid solutions taste perception questionnaire

We developed a brief taste perception questionnaire to evaluate participants’ taste perception of the liquid solution. This questionnaire included three items. (1) If you had to characterize the flavor of the previously used mouth-rinsing solution with a single taste category, how would you describe it? (2) Indicate the intensity of the taste left by the rinsing solution. (3) If the rinsing solution has left your mouth feeling rough and dry, rate the intensity of this sensation. The participants provided their responses using a horizontal sliding scale ranging from 0 to 10 or using five taste categories (sour, bitter, sweet, salty, and umami/savory).

### Procedure

#### Preliminary questionnaires

When participants initially enrolled in the experiment, they were given an online questionnaire to be completed to formalize their enrollment. This questionnaire requested information on (1) age, (2) gender (male, female, or nonbinary), (3) weight, and (4) height. The participants were then asked to complete our food consumption questionnaire and the STQ. Finally, the participants were asked about their lifestyle and dietary habits, which included several questions (see Appendix B in the Online Supplementary Material). All questionnaires were administered via the online platform Qualtrics (www.qualtrics.com) and were consistently completed at least 24 h prior to starting the experiment in the laboratory.

The participants were instructed to arrive at the scheduled day and time following a fasting period of at least 4 h. Upon their arrival, the participants were randomly assigned to one of two experimental groups. The experiment was conducted on a PC (Intel i5-7400, 3.00 GHz) using a routine programmed in PsychoPy (version v2022.2.5; Pierce, 2007) running on Windows 10 professional (64 bits) and presented on a 24-in. LCD monitor (AOC, Model G2460PF) with a refresh rate of 60 Hz. The stimuli were presented on a screen subtending ~ 9.0 degrees of the visual angle.

#### Experimental tasks

The participants were first instructed to complete a series of preliminary inquiries before beginning the experimental task: (1) estimation of the elapsed time (in hours) since their most recent meal consumption, (2) a description of the specific food consumed during that previous meal, and (3) self-reports of their current level of hunger. This subjective measure of hunger was recorded using a horizontal sliding scale ranging from 0 to 10, with 0 indicating a *complete absence of hunger* and 10 indicating an *extreme sensation of hunger*. The experimental tasks were administered immediately after this assessment. The procedure mirrored that used by Chen and colleagues (2016). Thus, the participants first completed a self-paced rating task in which they were instructed to rate the appeal of 90 food images presented one by one centered on the computer screen in random order. They responded by using a computer mouse to manipulate a 200-point horizontal slider, with -100 indicating *not at all appealing* and + 100 indicating *very much appealing*. The selected values on the visual analog scale were not visible to the participants.

The 45 food pictures from the two sets of images (sweet and savory foods) were ranked separately from highest to lowest rating scores. The top 25 images from each category were then selected as stimuli for the subsequent GNG task. The 50 selected pictures were divided into five subsets of ten pictures each. Within each subset, five images were sweet foods, and the other five were savory foods. This procedure ensured that the average rating scores were consistent across all image subsets. Next, the five subsets were randomly assigned as follows: three subsets to the go condition and one subset to the no-go condition; the remaining subset was not used in the task and served as a baseline (untrained condition).

Following the initial food-rating task, participants were instructed to perform an oral rinse using a 20-ml solution contained in a small glass placed adjacent to the computer screen. In addition to the solution, participants were provided with a separate glass of water (~ 40 ml) and a spittoon. The participants initiated the rinse by pressing the space bar, which triggered a countdown timer on the screen. They were requested to use the entire volume of the solution for 30 s of rinsing (Brala & Hagen, 1983) and to spit the solution into the provided container once the countdown was complete. Finally, the participants were given the option to perform a water rinse or drink before proceeding to the subsequent phases of the experiment.

The participants subsequently performed the cue-GNG task. In each trial, a food image was presented centrally on a white screen and remained visible for 1,000 ms. Next, 100 ms after the onset of the image presentation, a color frame, either blue or yellow, appeared and surrounded the displayed image for 300 ms. One specific color was designated for the go condition, whereas the other color was used for the no-go condition and counterbalanced across all participants. The participants were instructed to respond with both speed and accuracy by pressing the space bar on the computer keyboard in the go condition and to refrain from responding if the color frame signaled the no-go condition. To prevent temporal predictability, the intertrial interval (ITI) duration was jittered, ranging from 1,500 to 2,500 ms, in steps of 100 ms. Throughout the ITI, a fixation cross was consistently presented in the center of the screen. The whole task consisted of five blocks of 40 trials each, resulting in a total of 200 trials. The blocks were separated by 10-s breaks.

The participants underwent a preliminary training phase consisting of 16 trials before engaging in the GNG task. During this training phase, 16 images representing objects from different categories were presented. These images were equally (50%) and randomly assigned to both the "go" and "no-go" conditions.

After the GNG task, the participants again completed a self-report rating task in which they were instructed to rate the appeal of the 50 preselected food images (from the original 90 previously evaluated), of which 30 were assigned to the go condition, ten to the no-go condition, and the remaining ten to the untrained condition. Notably, half of the images belonged to the sweet category, and the other half belonged to the savory category.

At the end of the second evaluation task, the participants were asked again to (1) report their current hunger level, (2) complete the specifically designed questionnaire aimed at assessing the taste properties of the mouth-rinsing solution, and (3) express their opinion about what they thought the aim of this study could be. The GNG took approximately 15 min, whereas the entire experiment lasted approximately 30 min.

#### Statistical analysis

All of the data analyses were performed using IBM SPSS Statistics v.29. A mixed-design ANOVA was used to evaluate participants’ performance on the GNG task. The ANOVA incorporated two within-subject factors, *task condition* (go vs. no-go) and *food category* (sweet vs. savory), and a between-subject factor, *rinsing solution* (GA vs. placebo). To analyze the devaluation effect and for each food-training condition (untrained, go, and no-go), we computed the difference score by subtracting the average rating of the first evaluation – before the GNG task – from the average rating in the second assessment, which was conducted after completing the GNG task. A negative score corresponded to participants rating food images less appealing after undertaking the GNG task. To assess the comparability of the devaluation effect across the three *training conditions*, the two *food categories*, and the *rinsing solution* used, we conducted a repeated-measures mixed-design ANOVA. Finally, to assess the no-go devaluation effect across the different independent variables of the study, we first defined it as the difference in the devaluation scores between the no-go and the untrained conditions (no-go minus untrained) in the GNG task and then constructed a statistical model with *food category* and *rinsing solution* as factors.

## Results

Upon their arrival, the participants reported the number of hours of fasting since their last meal. There was no difference between the two groups [GA: 6.53 h (*SD*: 3.36); placebo: 7.73 h (*SD*: 4.13); *t*(98) = -1.56; *p* = 0.123; *d-cohen* = -0.32]. The self-reported hunger levels before and after the experiment indicated a statistically significant increase in hunger at the conclusion of the experiment [first hunger assessment: 6.9; second hunger assessment: 7.8; *F*(1,95) = 29.75; *p* < 0.001; *η*_p_^2^ = 0.24]. This increase was consistent across participants, as indicated by the lack of a significant interaction between *rinsing solution* and *hunger assessment* [*F*(1,95) = 0.70; *p* = 0.404; *η*_p_^2^ < 0.01] and the absence of a main effect of *rinsing solution* [*F*(1,95) = 0.03; *p* = 0.862; *η*_p_^2^ < 0.001]. We identified nine participants who suggested that the experiment might have been designed to induce devaluation through the GNG task (two in the GA and five in the placebo conditions) or that the rinsing procedure was designed to disrupt the evaluation of food images (one participant in each condition). However, further analyses revealed that excluding these participants did not significantly alter the results.^1^

### Questionnaires^2^

#### Food consumption questionnaire

There were no significant differences in food consumption patterns between the participants. Hence, self-reports on food consumption were similar for sweet [mean, GA: 5.13; placebo: 5.08; *t*(94) = 0.39; *p* = 0.697; *d* < -0.01; a 5-point score indicated a consumption frequency of *once per month*] and savory foods [mean, GA: 4.46; placebo solution: 4.51; *t*(94) = -0.45; *p* = 0.654; *d* = -0.09; a 4.5-point score indicated a frequency of consumption between *once every 2 weeks* and *once a month*].

#### Sweet test questionnaire

All participants exhibited comparable mid-range scores on the STQ, reflecting similar emotional sweetness dependence and control in relation to sweet food consumption [Factor 1, mean scores, GA: 3.80; placebo: 3.87; *t*(94) = -0.25, *p* = 0.805; *d* = -0.05; Factor 2, mean scores, GA: 3.17; placebo: 3.51; *t*(94) = -1.15, *p* = 0.254; *d* = -0.24].

#### Taste perception questionnaire

Most participants perceived the liquid solution as bitter (37/45 participants who rinsed their mouth with the GA solution and 46/52 participants who used the placebo solution rated it as bitter). A chi-square test revealed no significant differences in taste perception between the two groups [χ^2^(3) = 2.82; *p* = 0.420]. Moreover, taste intensity and “rough and dry” sensation were also similar between the two groups [*t*(95) = 1.72, *p* = 0.088; *d* = 0.35; *t*(95) = 0.63, *p* = 0.533; *d* = 0.12].

### Go/no-go results

The results revealed the expected main effect of the *GNG condition* [*F*(1,95) = 138.53; *p* < 0.001; *η*_p_^2^ = 0.59], reflecting a much greater accuracy on the go trials [99.7% (*SD* = 0.6)] than on the no-go trials [89.4% (*SD* = 8.9)] (see Table 1). No significant main effects were detected for *food category* [*F*(1,95) = 0.01; *p* = 0.945; *η*_p_^2^ < 0.001] or *rinsing solution* [*F*(1,95) = 3.02; *p* = 0.086; *η*_p_^2^ = 0.03]. The interaction effect between the GNG condition and rinsing solution did not reach statistical significance [*F*(1,95) = 3.00; *p* = 0.086; *η*_p_^2^ = 0.03]. Similarly, the three-way interactions among the GNG condition, food category, and rinsing solution was not statistically significant [*F*(1,95) = 2.34; *p* = 0.130; *η*_p_^2^ = 0.02], nor were any of the other interaction terms (all *p*-values > 0.1).

**Table 1:** Participants’ accuracy scores in the GNG task

	**Food categories**	**Go**		**NoGo**	
		Accuracy (%)	SEM	Accuracy (%)	SEM
**GA**	*Savory*	99.8	0.1	88.4	1.5
	*Sweet*	99.6	0.1	87.0	1.6
**placebo**	*Savory*	99.8	0.1	89.9	1.4
	*Sweet*	99.7	0.1	91.8	1.4

The mean average scores and standard errors of the means (SEMs) were calculated for the rinsing solution (gymnemic acid vs. placebo), food category (savory vs. sweet) and GNG training conditions (Go vs. NoGo)

### Food devaluation

**Fig. 1:**
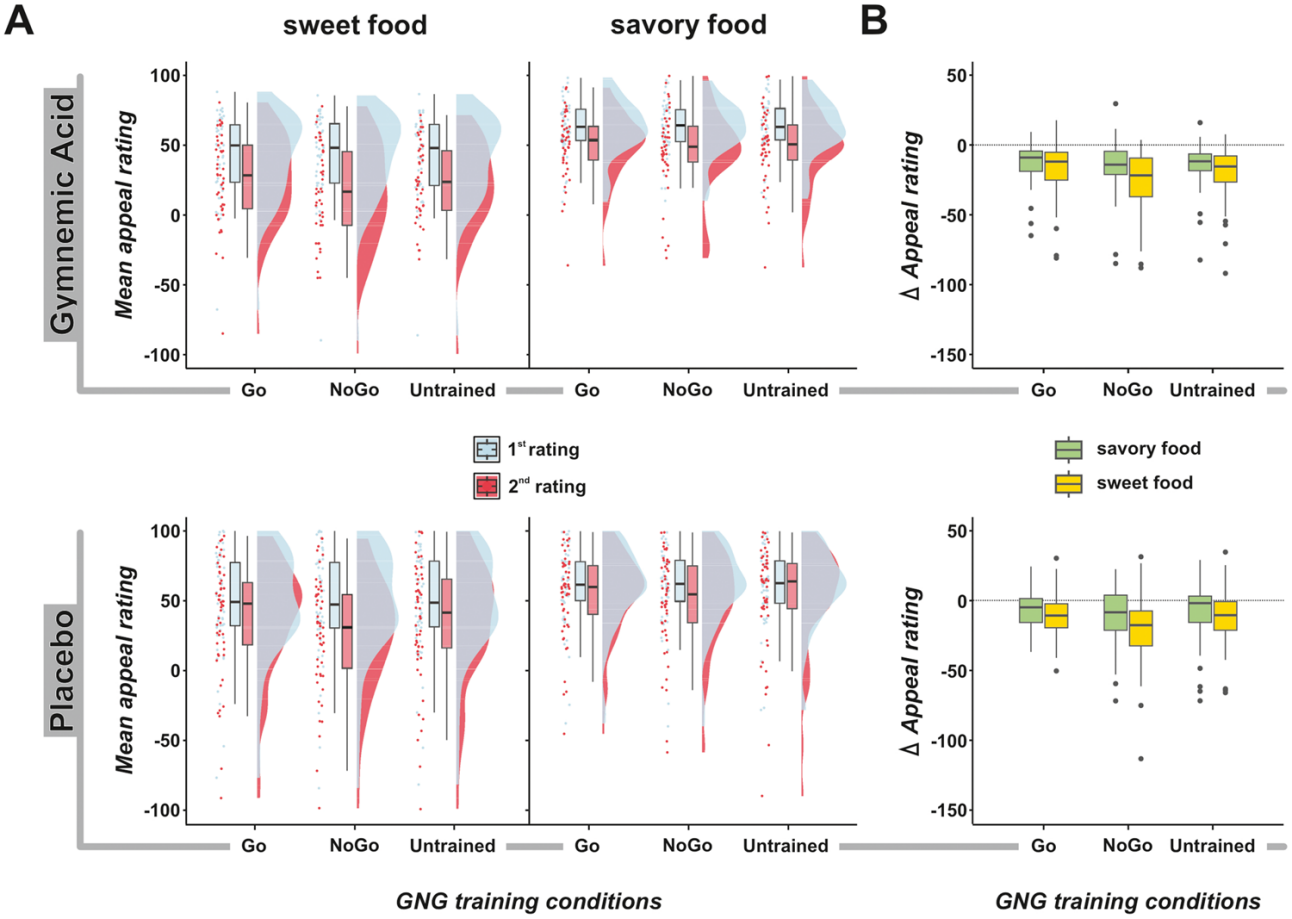
Food appeal ratings and devaluation effects. (**A**) The distribution of mean appeal ratings for first and second ratings (before and after the go/no-go task, respectively), separated by *rinsing solution* (gymnemic acid (GA) and placebo), *food category* (sweet and savory), and GNG *training conditions*. (**B**) Boxplots showing the difference scores (∆ Appeal: second rating minus first rating) for the different conditions of the experiment. On average, all foods were rated as less appealing relative to the baseline (first rating), with this rating change being significantly larger for no-go training conditions and especially for sweet foods

The total difference scores for the three training conditions were as follows: untrained, *M* = -13.8 (*SD* = 16.3); go, *M* = -11.1 (*SD* = 14.7); and no-go, *M* = -18.7 (*SD* = 18.6) (see Fig. 1 and Table 2). The difference score was negative in all training conditions, indicating that food items were all scored as less appealing during the second evaluation, validating and reproducing the results obtained in previous studies (Chen et al., 2016). The results revealed main effects for both *training conditions* [*F*(2,190) = 14.98; *p* < 0.001; *η*_p_^2^ = 0.14] and *food category* [*F*(1,95) = 15.81; *p* < 0.001; *η*_p_^2^ = 0.14], as well as a significant interaction between *training conditions* and *food category* [*F*(2,95) = 4.63; *p* = 0.014; *η*_p_^2^ = 0.05]. Furthermore, a significant main effect of rinsing solution was observed [*F*(1,95) = 4.38; *p* = 0.039; *η*_p_^2^ = 0.04], indicating greater general food devaluation in participants in the GA condition. However, the three-way interaction (training conditions × food category × rinsing solution) was not statistically significant [*F*(2,190) = 0.06; *p* = 0.924; *η*_p_^2^ < 0.01].

**Table 2:** Mean average devaluation effect (2nd evaluation – 1st evaluation) and standard error of the mean (SEM) for the rinsing solution (gymnemic acid vs. placebo), food category (savory vs. sweet) and GNG training conditions (Go vs. NoGo)

	**savory foods**						**sweet foods**					
	*untrained*		*Go*		*NoGo*		*untrained*		*Go*		*NoGo*	
	M	SEM	M	SEM	M	SEM	M	SEM	M	SEM	M	SEM
**GA**	-14.4	2.5	-12.4	2.2	-15.6	3.1	-20.5	3.1	-17.0	3.2	-27.7	3.7
**placebo**	-7.9	2.9	-6.3	2.0	-10.9	2.7	-13.6	2.8	-10.2	2.3	-21.1	3.5

We then conducted separate post hoc comparisons for savory and sweet foods between the different GNG training conditions. The summarized descriptive results are presented in Table 2. For sweet foods, devaluation was significantly greater in the no-go condition than in the untrained condition [*t*(96) = -3.57; *p* = 0.001; *d* = -0.36] and in the go condition [*t*(96) = -5.44; *p* < 0.001; *d* = -0.55]. Additionally, the devaluation was greater for the untrained condition than for the go condition [*t*(96) = -2.38; *p* = 0.019; *d* = -0.24]. With savory foods, only the devaluation for the no-go condition was greater than that for the go condition [*t*(96) = -2.16; *p* = 0.033; *d* = -0.22]. Finally, the no-go devaluation effect (no-go minus untrained GNG conditions) was greater for sweet foods, as indicated by the main effect of food category [sweet foods: -7.4 (*SD* = 20.5); savory foods: -2.2 (*SD* = 18.9), *F*(1,95) = 4.10; *p* = 0.046; *η*_p_^2^ = 0.04]. In contrast, neither the rinsing solution [*F*(1,95) = 0.124; *p* = 0.725; *η*_p_^2^ < 0.01] nor the interaction term [*F*(1,95) = 0.09; *p* = 0.766; *η*_p_^2^ < 0.01] reached statistical significance.

## Discussion

In the present study, we tested value updating and inhibitory accounts as plausible theoretical explanations for the no-go devaluation effect. To this end, our participants completed a rating on the appeal of either savory or sweet foods twice – before and after completing the GNG task. Crucially, participants were asked to rinse their mouths with an undisclosed liquid solution immediately after the first food rating was completed. We employed a rigorous double-blind design in which one group of participants rinsed their mouths with GA (Sanematsu et al., 2014), whereas another group of participants received a placebo solution. The purpose of GA rinsing was to selectively block the activation of the neural circuits involved in sweet-taste processing at the sensory level (Stice & Yokum, 2018) to alter the expected pleasantness of the food images presented during the GNG task (Stice et al., 2017). The results indicated that participants who rinsed their mouths with GA exhibited a stronger overall decrease in food evaluations from pre to post training of all the food items. Interestingly, the GNG training resulted in a considerably greater no-go devaluation of sweet compared with savory foods. We replicated the findings from previous studies on GNG training; however, we found that GA did not modulate the no-go devaluation effect for sweet food but instead uniformly reduced the perceived value of all food items during the second evaluation, reflecting a regression to the mean.

The no-go devaluation effect refers to the empirical phenomenon in which no-go items in the GNG task are consistently rated as less appealing than both untrained and go items after task completion (Chen et al., 2016, 2018a, 2018b). This effect has been robustly replicated across numerous studies (McGreen et al., 2024b) and observed in both normal-weight and obese populations (Chen et al., 2016; Chen, Veling, De Vries, et al., 2018a). The persistence of the devaluation effect has been shown to be influenced by the consistency and the conveyed meaning of the food category paired with no-go responses (Serfas et al., 2017) and is highly sensitive to alterations in the experimental protocol (Liu et al., 2022). Although there is no consensus on the conceptualization of food valuation, it is generally assumed that eating behavior is frequently driven by strong affective reactions and Pavlovian conditioned responses to food cues (Tzavella & Chambers, 2023). Our results suggest that GA did not moderate the no-go devaluation effect. Interference with sweet-associated reward expectancy induced only a general food devaluation effect, likely due to regression to the mean. Notably, the significantly greater devaluation effect of sweet foods than of savory foods presents a challenge to both theoretical accounts under consideration. According to inhibitory theory, similar response inhibition is elicited across all no-go trials, irrespective of the food category, a prediction that our results do not support. Similarly, the value-updating account suggests that greater discrepancies between stimulus values and inaction commitments lead to increased conflicts and consequently greater devaluation (Chen et al., 2016). However, our findings seem to challenge this view as well. Specifically, although the initial appeal of savory foods far exceeded that of sweet foods (Table 3), a significantly stronger no-go devaluation effect was observed for sweet items.

**Table 3:** The average evaluation score obtained in the initial food evaluation task

	**food categories**	untrained	Go	NoGo	*F* value *df*(2,88)	*p* value
**GA**	*savory*	62.4	62.5	62.1	0.81	0.45
	sweet	41.6	42.2	42.2	0.69	0.51
**placebo**	*savory*	59.9	60.2	60.4	1.03	0.36
	sweet	48.1	47.7	47.6	0.97	0.38
*GA vs. placebo*					*F*(1,95) = 0.12	0.73
*savory vs. sweet*					*F*(1,95) = 27.5	< 0.001

The results obtained indicate that the program effectively distributed the items uniformly across all three training GNG conditions (untrained, Go and NoGo). Notably, i) the scores assigned were consistent between the two groups of participants [gymnemic acid (GA) and placebo], and ii) the values attributed to sweet foods were significantly lower than those attributed to savory foods

A possible explanation for these results within the value-updating framework is that explicit liking, as measured by food appeal ratings, does not perfectly correspond to implicit wanting (see Pool et al., 2016, for a review). Thus, the no-go devaluation effect may largely reflect the modulation of Pavlovian-conditioned responses occurring during GNG training. This can be interpreted as a reduction in the incentive salience – or wanting – of food items associated with the no-go cue (Berridge, 2007; Morales & Berridge, 2020). Similarly, altering food reward expectancy with GA does not influence these automatic response biases (Berridge & Aldridge, 2008; Berridge & O’Doherty, 2014). Indeed, wanting sweet foods is strongly influenced by liking but has also been found to depend on other factors, such as social norms, the presence of others during a meal, and metabolic homeostasis (Berridge, 2009). Thus, the ingestion of sweets results in a decrease in its pleasantness rating, which is attributed to the metabolic effects of sugar, a phenomenon known as *negative alliesthesia* (Frankham et al., 2005; Hetherington et al., 1989).

Overall, a broad interpretation of our findings aligns with previous studies suggesting that the GNG task can be conceptualized as a tool for cognitive training aimed at enhancing self-control, in which self-control is assumed to be a process involving value-based decision-making (Berkman et al., 2017). By reframing the GNG task as a decision-making paradigm rather than merely an inhibitory paradigm, it is reasonable to infer that GNG training could facilitate changes in the subjective value attributed to food (Rangel et al., 2008). Despite the inherent simplicity of the GNG task, in which individuals respond to some items and withhold their response to others, there is substantial evidence supporting the power of such a task to reduce the value of food- (Allom et al., 2016; McGreen et al., 2024a; Turton et al., 2016), alcohol- (Houben et al., 2011, 2012), or smoking-related cues (Scholten et al., 2019). With practice, it is possible to change the value of no-go items and consequently food preferences. One example is the use of ultra-processed foods as no-go items to reduce their consumption (Veling et al., 2022).

Preferences for sweet taste are universal, although there are large interindividual variations in the preferred intensity and type of sweet foods consumed (see Drewnowski et al., 2012, for a review). The existing evidence suggests that the habitual intake of palatable ultra-processed foods in the current obesogenic environment has been associated with a steady increase in the prevalence of obesity in recent decades (Di Cesare et al., 2016; Lieberman, 2006). Similarly, the low cost and widespread availability of energy-dense sweeteners in food supplies are directly related to the increasing intake of added sugars, indirectly contributing to the obesity pandemic (Darmon & Drewnowski, 2015; Drewnowski et al., 2012). Successful intervention plans are needed to reverse this situation since simply asking people to actively inhibit their drive for hedonic satisfaction has not proven effective. Instead, strategies that focus on changing the value of food, such as emphasizing the expectation of a positive taste experience when consuming healthy foods (Turnwald et al., 2019), engaging in a simple GNG task that pairs unhealthy foods with inaction, or drawing attention to health-related aspects of foods during decision-making (Hare et al., 2011), have proven to be promising avenues for promoting the adoption of nutritious eating habits.

## Supplementary Information

Below is the link to the electronic supplementary material.

Supplementary file1 (DOCX 17 KB)

## Data Availability

The data and materials for the experiment, as well as the script of the experimental task are available at: 10.17605/OSF.IO/9RB42.

## Footnotes

1 The results after removal of these participants were very similar, with persistent significant main effects for *training* [*F*(2,172) = 8.57; *p* < 0.001; *η*_p_^2^ = 0.091] and *food category* [*F*(1,86) = 9.23; *p* = 0.003; *η*_p_^2^ = 0.097]; a significant interaction between *training* and *food category* [*F*(2,172) = 3.62; *p* = 0.035; *η*_p_^2^ = 0.04], and a significant main effect of *rinsing solution* [F(1,86) = 4.17; *p* = 0.044; *η*_p_^2^ = 0.046].

2 Data from one participant were missing from all questionnaires completed during the enrollment process. Nevertheless, we decided to retain the participant’s dataset for subsequent experimental data analysis.

## Code availability

The scripts of the experimental task and or generated for the statistical analysis are available at: 10.17605/OSF.IO/9RB42.

## References

[CR1] Allom, V., Mullan, B., & Hagger, M. (2016). Does inhibitory control training improve health behaviour? *A Meta-Analysis. Health Psychology Review,**10*(2), 168–186. 10.1080/17437199.2015.1051078 26058688 10.1080/17437199.2015.1051078

[CR2] Aulbach, M. B., Knittle, K., & Haukkala, A. (2019). Implicit process interventions in eating behaviour: A meta-analysis examining mediators and moderators. *Health Psychology Review,**13*(2), 179–208. 10.1080/17437199.2019.1571933 30676235 10.1080/17437199.2019.1571933

[CR3] Berkman, E. T., Hutcherson, C. A., Livingston, J. L., Kahn, L. E., & Inzlicht, M. (2017). Self-Control as Value-Based Choice. *Current Directions in Psychological Science,**26*(5), 422–428. 10.1177/0963721417704394 29335665 10.1177/0963721417704394 PMC5765996

[CR4] Berridge, K. C. (2007). The debate over dopamine’s role in reward: The case for incentive salience. *Psychopharmacology (Berl),**191*(3), 391–431. 10.1007/s00213-006-0578-x 17072591 10.1007/s00213-006-0578-x

[CR5] Berridge, K. C. (2009). “Liking” and “wanting” food rewards: Brain substrates and roles in eating disorders. *Physiology and Behavior,**97*(5), 537–550. 10.1016/j.physbeh.2009.02.044 19336238 10.1016/j.physbeh.2009.02.044 PMC2717031

[CR6] Berridge, K. C., & Aldridge, J. W. (2008). Decision utility, the brain, and pursuit of hedonic goals. *Social Cognition,**26*(5), 621–646. 10.1521/soco.2008.26.5.621 20198128 10.1521/soco.2008.26.5.621 PMC2830022

[CR7] Berridge, K. C., & O’Doherty, J. P. (2014). *Chapter 18 - From Experienced Utility to Decision Utility* (P. W. Glimcher & E. B. T.-N. (Second E. Fehr (eds.); pp. 335–351). Academic Press. 10.1016/B978-0-12-416008-8.00018-8

[CR8] Blechert, J., Meule, A., Busch, N. A., & Ohla, K. (2014). Food-pics: An image database for experimental research on eating and appetite. *Frontiers in Psychology*, *5*(JUN), 1–10. 10.3389/fpsyg.2014.00617 10.3389/fpsyg.2014.00617 PMC4067906 25009514

[CR9] Botvinik-Nezer, R., Salomon, T., & Schonberg, T. (2020). Enhanced Bottom-Up and Reduced Top-Down fMRI Activity Is Related to Long-Lasting Nonreinforced Behavioral Change. *Cerebral Cortex,**30*(3), 858–874. 10.1093/cercor/bhz132 31408106 10.1093/cercor/bhz132 PMC7132905

[CR10] Brala, P. M., & Hagen, R. L. (1983). Effects of sweetness perception and caloric value of a preload on short term intake. *Physiology and Behavior,**30*(1), 1–9. 10.1016/0031-9384(83)90030-6 6836034 10.1016/0031-9384(83)90030-6

[CR11] Chen, Z., Veling, H., De Vries, S. P., Bijvank, B. O., Janssen, I. M. C., Dijksterhuis, A., & Holland, R. W. (2018a). Go/No-Go Training Changes Food Evaluation in Both Morbidly Obese and Normal-Weight Individuals. *Journal of Consulting and Clinical Psychology,**86*(12), 980–990. 10.1037/ccp0000320 30507224 10.1037/ccp0000320

[CR12] Chen, Z., Veling, H., Dijksterhuis, A., & Holland, R. W. (2016). How does not responding to appetitive stimuli cause devaluation: Evaluative conditioning or response inhibition? *Journal of Experimental Psychology: General,**145*(12), 1687–1701. 10.1037/xge0000236 27736134 10.1037/xge0000236

[CR13] Chen, Z., Veling, H., Dijksterhuis, A., & Holland, R. W. (2018b). Do impulsive individuals benefit more from food go/no-go training? Testing the role of inhibition capacity in the no-go devaluation effect. *Appetite,**124*, 99–110. 10.1016/j.appet.2017.04.024 28442335 10.1016/j.appet.2017.04.024

[CR14] Darmon, N., & Drewnowski, A. (2015). Contribution of food prices and diet cost to socioeconomic disparities in diet quality and health: A systematic review and analysis. *Nutrition Reviews,**73*(10), 643–660. 10.1093/nutrit/nuv027 26307238 10.1093/nutrit/nuv027 PMC4586446

[CR15] Di Cesare, M., Bentham, J., Stevens, G. A., Zhou, B., Danaei, G., Lu, Y., Bixby, H., Cowan, M. J., Riley, L. M., Hajifathalian, K., Fortunato, L., Taddei, C., Bennett, J. E., Ikeda, N., Khang, Y. H., Kyobutungi, C., Laxmaiah, A., Li, Y., Lin, H. H., … Cisneros, J. Z. (2016). Trends in adult body-mass index in 200 countries from 1975 to 2014: A pooled analysis of 1698 population-based measurement studies with 19.2 million participants. *The Lancet*, *387*(10026), 1377–1396. 10.1016/S0140-6736(16)30054-X 10.1016/S0140-6736(16)30054-X PMC7615134 27115820

[CR16] Diamond, A. (2013). Executive functions. *Annual Review of Psychology,**64*, 135–168. 10.1146/annurev-psych-113011-143750 23020641 10.1146/annurev-psych-113011-143750 PMC4084861

[CR17] Drewnowski, A., Mennella, J. A., Johnson, S. L., & Bellisle, F. (2012). Sweetness and food preference. *Journal of Nutrition,**142*(6), 1142–1148. 10.3945/jn.111.149575 10.3945/jn.111.149575 PMC3738223 22573785

[CR18] Erdfelder, E., Faul, F., Buchner, A., & Lang, A. G. (2009). Statistical power analyses using G*Power 3.1: Tests for correlation and regression analyses. *Behavior Research Methods*, *41*(4), 1149–1160. 10.3758/BRM.41.4.1149 10.3758/BRM.41.4.1149 19897823

[CR19] Frankham, P., Gosselin, C., & Cabanac, M. (2005). Diet induced weight loss accelerates onset of negative alliesthesia in obese women. *BMC Public Health,**5*, 1–8. 10.1186/1471-2458-5-112 16232316 10.1186/1471-2458-5-112 PMC1266380

[CR20] Goolsby, B. A., Shapiro, K. L., & Raymond, J. E. (2009). Distractor devaluation requires visual working memory. *Psychonomic Bulletin and Review,**16*(1), 133–138. 10.3758/PBR.16.1.133 19145023 10.3758/PBR.16.1.133

[CR21] Hare, T. A., Malmaud, J., & Rangel, A. (2011). Focusing attention on the health aspects of foods changes value signals in vmPFC and improves dietary choice. *Journal of Neuroscience,**31*(30), 11077–11087. 10.1523/JNEUROSCI.6383-10.2011 21795556 10.1523/JNEUROSCI.6383-10.2011 PMC6623079

[CR22] Hawkes, C., Smith, T. G., Jewell, J., Wardle, J., Hammond, R. A., Friel, S., Thow, A. M., & Kain, J. (2015). Smart food policies for obesity prevention. *The Lancet,**385*(9985), 2410–2421. 10.1016/S0140-6736(14)61745-1 10.1016/S0140-6736(14)61745-1 25703109

[CR23] Hetherington, M., Rolls, B. J., & Burley, V. J. (1989). The time course of sensory-specific satiety. *Appetite,**12*(1), 57–68. 10.1016/0195-6663(89)90068-8 2719473 10.1016/0195-6663(89)90068-8

[CR24] Houben, K., & Aulbach, M. (2023). Is there a difference between stopping and avoiding? A review of the mechanisms underlying Go/No-Go and Approach-Avoidance training for food choice. *Current Opinion in Behavioral Sciences*, *49*, 101245. 10.1016/j.cobeha.2022.101245

[CR25] Houben, K., Havermans, R. C., Nederkoorn, C., & Jansen, A. (2012). Beer à no-go: Learning to stop responding to alcohol cues reduces alcohol intake via reduced affective associations rather than increased response inhibition. *Addiction,**107*(7), 1280–1287. 10.1111/j.1360-0443.2012.03827.x 22296168 10.1111/j.1360-0443.2012.03827.x

[CR26] Houben, K., & Jansen, A. (2015). Chocolate equals stop: Chocolate-specific inhibition training reduces chocolate intake and go associations with chocolate. *Appetite,**87*, 318–323. 10.1016/j.appet.2015.01.005 25596041 10.1016/j.appet.2015.01.005

[CR27] Houben, K., Nederkoorn, C., Wiers, R. W., & Jansen, A. (2011). Resisting temptation: Decreasing alcohol-related affect and drinking behavior by training response inhibition. *Drug and Alcohol Dependence,**116*(1–3), 132–136. 10.1016/j.drugalcdep.2010.12.011 21288663 10.1016/j.drugalcdep.2010.12.011

[CR28] Kampov-Polevoy, A. B., Alterman, A., Khalitov, E., & Garbutt, J. C. (2006). Sweet preference predicts mood altering effect of and impaired control over eating sweet foods. *Eating Behaviors,**7*(3), 181–187. 10.1016/j.eatbeh.2005.09.005 16843219 10.1016/j.eatbeh.2005.09.005

[CR29] Lieberman, L. S. (2006). Evolutionary and anthropological perspectives on optimal foraging in obesogenic environments. *Appetite,**47*(1), 3–9. 10.1016/j.appet.2006.02.011 16806580 10.1016/j.appet.2006.02.011

[CR30] Liu, H., Holland, R. W., Blechert, J., Quandt, J., & Veling, H. (2022). Devaluation of NoGo stimuli is both robust and fragile. *Cognition and Emotion*. 10.1080/02699931.2022.2067132 35467479 10.1080/02699931.2022.2067132

[CR31] McGreen, J., Kemps, E., & Tiggemann, M. (2024a). The effectiveness of Go/No-Go and Stop-Signal training in reducing food consumption and choice: A systematic review and meta-analysis. *Appetite*, *195*, 107215. 10.1016/j.appet.2024.107215 10.1016/j.appet.2024.107215 38309625

[CR32] McGreen, J., Kemps, E., & Tiggemann, M. (2024b). The effectiveness of Go/No-Go and Stop-Signal training in reducing food consumption and choice: A systematic review and meta-analysis. *Appetite*, *195*, 107215. 10.1016/j.appet.2024.107215 10.1016/j.appet.2024.107215 38309625

[CR33] Morales, I., & Berridge, K. C. (2020). ‘Liking’ and ‘wanting’ in eating and food reward: Brain mechanisms and clinical implications. *Physiology and Behavior,**227*, 113152. 10.1016/j.physbeh.2020.113152 32846152 10.1016/j.physbeh.2020.113152 PMC7655589

[CR34] Pool, E., Sennwald, V., Delplanque, S., Brosch, T., & Sander, D. (2016). Measuring wanting and liking from animals to humans: A systematic review. *Neuroscience and Biobehavioral Reviews,**63*, 124–142. 10.1016/j.neubiorev.2016.01.006 26851575 10.1016/j.neubiorev.2016.01.006

[CR35] Rangel, A., Camerer, C., & Montague, P. R. (2008). A framework for studying the neurobiology of value-based decision making. *Nature Reviews Neuroscience,**9*(7), 545–556. 10.1038/nrn2357 18545266 10.1038/nrn2357 PMC4332708

[CR36] Sanematsu, K., Kusakabe, Y., Shigemura, N., Hirokawa, T., Nakamura, S., Imoto, T., & Ninomiya, Y. (2014). Molecular mechanisms for sweet-suppressing effect of gymnemic acids. *Journal of Biological Chemistry,**289*(37), 25711–25720. 10.1074/jbc.M114.560409 25056955 10.1074/jbc.M114.560409 PMC4162174

[CR37] Scholten, H., Granic, I., Chen, Z., Veling, H., & Luijten, M. (2019). Do smokers devaluate smoking cues after go/no-go training? *Psychology and Health,**34*(5), 609–625. 10.1080/08870446.2018.1554184 30693789 10.1080/08870446.2018.1554184

[CR38] Schonberg, T., & Katz, L. N. (2020). A Neural Pathway for Nonreinforced Preference Change. *Trends in Cognitive Sciences,**24*(7), 504–514. 10.1016/j.tics.2020.04.002 32430228 10.1016/j.tics.2020.04.002

[CR39] Serfas, B. G., Florack, A., Büttner, O. B., & Voegeding, T. (2017). What does it take for sour grapes to remain sour? Persistent effects of behavioral inhibition in go/no-go tasks on the evaluation of appetitive stimuli. *Motivation Science,**3*(1), 1–18. 10.1037/mot0000051

[CR40] Stice, E., & Yokum, S. (2018). Effects of gymnemic acids lozenge on reward region response to receipt and anticipated receipt of high-sugar food. *Physiology and Behavior,**194*, 568–576. 10.1016/j.physbeh.2018.07.012 30031752 10.1016/j.physbeh.2018.07.012

[CR41] Stice, E., Yokum, S., & Gau, J. M. (2017). Gymnemic acids lozenge reduces short-term consumption of high-sugar food: A placebo controlled experiment. *Journal of Psychopharmacology,**31*(11), 1496–1502. 10.1177/0269881117728541 28944714 10.1177/0269881117728541

[CR42] Turnwald, B. P., Bertoldo, J. D., Perry, M. A., Policastro, P., Timmons, M., Bosso, C., Connors, P., Valgenti, R. T., Pine, L., Challamel, G., Gardner, C. D., & Crum, A. J. (2019). Increasing Vegetable Intake by Emphasizing Tasty and Enjoyable Attributes: A Randomized Controlled Multisite Intervention for Taste-Focused Labeling. *Psychological Science,**30*(11), 1603–1615. 10.1177/0956797619872191 31577177 10.1177/0956797619872191 PMC6843749

[CR43] Turton, R., Bruidegom, K., Cardi, V., Hirsch, C. R., & Treasure, J. (2016). Novel methods to help develop healthier eating habits for eating and weight disorders: A systematic review and meta-analysis. *Neuroscience and Biobehavioral Reviews,**61*, 132–155. 10.1016/j.neubiorev.2015.12.008 26695383 10.1016/j.neubiorev.2015.12.008

[CR44] Tzavella, L., & Chambers, C. D. (2023). Explicit and Implicit Devaluation Effects of Food-Specific Response Inhibition Training. *Journal of Cognition,**6*(1), 1–20. 10.5334/joc.256 36721799 10.5334/joc.256 PMC9854316

[CR45] Veling, H., Becker, D., Liu, H., Quandt, J., & Holland, R. W. (2022). How go/no-go training changes behavior: A value-based decision-making perspective. *Current Opinion in Behavioral Sciences,**47*, 101206. 10.1016/j.cobeha.2022.101206

[CR46] Veling, H., Chen, Z., Tombrock, M. C., Verpaalen, I. A. M., Schmitz, L. I., Dijksterhuis, A., & Holland, R. W. (2017). Training impulsive choices for healthy and sustainable food. *Journal of Experimental Psychology: Applied,**23*(2), 204–215. 10.1037/xap0000112 28150960 10.1037/xap0000112

[CR47] Ventura, A. K., & Worobey, J. (2013). Early influences on the development of food preferences. *Current Biology,**23*(9), R401–R408. 10.1016/j.cub.2013.02.037 23660363 10.1016/j.cub.2013.02.037

[CR48] Yang, Y., Shields, G. S., Wu, Q., Liu, Y., Chen, H., & Guo, C. (2019). Cognitive training on eating behaviour and weight loss: A meta-analysis and systematic review. *Obesity Reviews,**20*(11), 1628–1641. 10.1111/obr.12916 31353774 10.1111/obr.12916

