# Supplementary material for: Tuning the value of sweet food: Blocking sweet taste receptors increases the devaluation effect in a go/no-go task
Source: Psychon Bull Rev. 2025 Feb 25;32(4):1785–94. doi: 10.3758/s13423-025-02666-w (PMC12325463; doi:10.3758/s13423-025-02666-w)
Supplement: Supplementary file 1 — Supplementary file1 (DOCX 17 KB) [file 13423_2025_2666_MOESM1_ESM.docx]

**Appendix A**: Ninety food images from the Food Pictures Extended database (Blechert et al., 2014) were used, half depicting palatable sweet foods and half depicting palatable savory foods. The items were listed alphabetically.

**Sweet foods**: apples; banana; bowl of muesli (granola); breakfast cereals; brownie with nuts; cantaloup; cheese cake with strawberries and crumbles; cherries; chocolate cake; chocolate cake; chocolate cookies; chocolate croissants; chocolate muffin; chocolate; popsicle with nuts; chocolate popsicles; cookie mix; cookies filled with chocolate cream; croissants; donut; donut with chocolate sprinkles; figs; filled chocolates; German krapfen; grapes (white); ice cream; ice cream sandwiches; ice cream with chocolate beans; lemon cake; muesli bar (oatmeal); muffins; oranges; pastries; peach; pear; raspberry cake; shortbreads; slice of bread topped with chocolate cream; some bars of chocolate (stacked); strawberries; strawberry ice cream cone; toast with jam; waffle; watermelon; wildberries mix; wine gum

**Savory foods**: almonds; bowl of rice; Bread roll; brie cheese; cheese and cold meat platter; cheese burger, french fries and cola; chips; chips (pringles); crackers; crisp bread; with cottage cheese; doner kebab; fillet of pork, grilled; fish sticks; french fries; french fries and chicken drumsticks; fried egg; glass of gherkins; ham sandwich; ham sandwich with chips; Hamburger with bacon; hot dog; lasagna; mixed vegetables; olives; pasta bake; pasta with bacon; pizza (ham and mushrooms); popcorn; Potatoe Wedges; rice waffles; Roast Beef; roasted chicken; salad plate; salami sausage; salmon with vegetables, grilled; smoked ham / bacon; spaghetti with pesto; Spaghetti with tomato sauce; sushi rolls; tomato and mozzarella; tomatoes; tortilla chips; trout with potatoes, pan fried; Viennese Schnitzel; walnut.

**Appendix B**:

Living and eating habits, questions: i) Who do you live with? ii) Who is responsible for cooking at home?, iii) Where do you typically have your meals? iv) Are you currently following any specific diet, and if so, which one? v) What types of food do you typically eat? vi) Do you have any known food intolerances? vii) How much time has passed since your last meal? viii) How hungry do you feel at the moment?
